# Focally Appearing Activation Map of a Reentrant Tachycardia Using a New Coherent Mapping Tool

**DOI:** 10.19102/icrm.2021.120302

**Published:** 2021-03-15

**Authors:** Abdul Q. Haji, Asim Kichloo, John G. Symons, Khalil Kanjwal

**Affiliations:** ^1^Walter Reed National Military Medical Center, Bethesda, MD, USA; ^2^VA Medical Center, Martinsburg, WV, USA; ^3^Division of Internal Medicine, Central Michigan University, Mount Pleasant, MI, USA; ^4^Division of Cardiology, McLaren Greater Lansing Hospital, Michigan State University, Lansing, MI, USA

**Keywords:** Ablation, AVRT, coherent, mapping, three-dimensional

## Abstract

Here, we discuss mapping of an atrioventricular reciprocating tachycardia (AVRT) using color-coding and a coherence module of the CARTO^®^ mapping system (Biosense Webster, Diamond Bar, CA, USA). AVRT is a reentry tachycardia and, when the atrial exit site of the arrhythmia circuit was mapped in this case, it appeared to have a focal centrifugal activation pattern as depicted by coherent mapping.

## Introduction

Three-dimensional (3D) activation mapping is extensively used during the mapping and ablation of various focal and reentrant arrhythmias. Accessory pathway– mediated tachycardia is a reentrant arrhythmia and the utility of electroanatomic mapping (EAM) using an activation map with color-coding is limited during the mapping and ablation of such a tachycardia.^1^ We describe a case in which activation mapping of the atrial exit-site accessory pathway conduction and coherent mapping using the CARTO^®^ 3 system (Biosense Webster, Diamond Bar, CA, USA) were performed and ablation was completed without any use of fluoroscopy.

## Care presentation

**Figure 1:**
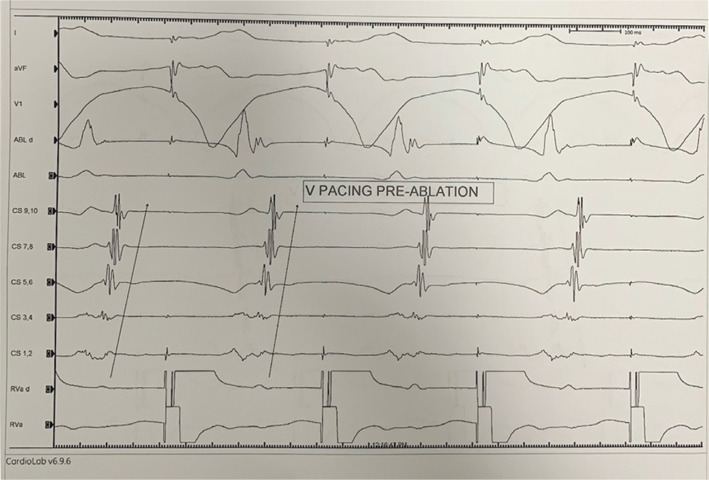
Ventricular pacing during sinus rhythm demonstrating eccentric retrograde conduction.

**Figure 2:**
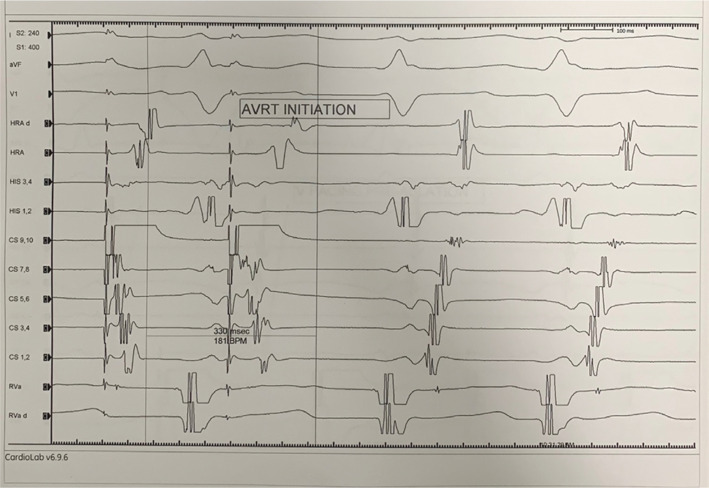
During AVRT, the retrograde atrial activation was eccentric, consistent with a left-side free wall accessory pathway.

A 27-year-old male with a history of recurrent palpitations was evaluated in the electrophysiology clinic. He had a documented episode of narrow complex tachycardia on a cardiac monitor. The patient was offered an electrophysiology study (EPS) and underwent EPS and catheter ablation in the postabsorptive state, under general anesthesia. Surface electrocardiography (ECG) and bipolar electrograms were continuously monitored using the CardioLab™ Electrophysiology Recording System (GE Healthcare, Chicago, IL, USA), sampled at 1 kHz and bandpass-filtered at 30 to 300 Hz. A multielectrode mapping catheter (PentaRay Nav; Biosense Webster) and a 3.5-mm open-irrigated-tip ablation catheter (Thermo-Cool; Biosense Webster) were placed through sheaths into the atrial chamber of interest. Then, the 3D fast anatomical map of the cardiac structures was acquired. The catheters were advanced and placed in the right atrium, His bundle, right ventricle, and coronary sinus. During the EP study, the retrograde conduction was eccentric **(Figure 1)**. The tachycardia was induced easily with an eccentric retrograde atrial activation pattern **(Figure 2)**. Finally, the diagnosis of left-side lateral atrioventricular reciprocating tachycardia (AVRT) was made using various maneuvers described previously.^2^

AVRT was induced with programmed stimulation from the proximal and distal coronary sinuses. After inducing sustained AVRT, 3D EAM was performed. A transseptal puncture under intracardiac echocardiography (ICE) guidance was performed to access the left atrium and intravenous heparin was administered to maintain an activated clotting time of between 300 and 350 seconds. Activation mapping was performed after inducing (AVRT) and during RV pacing.

**Figure 3:**
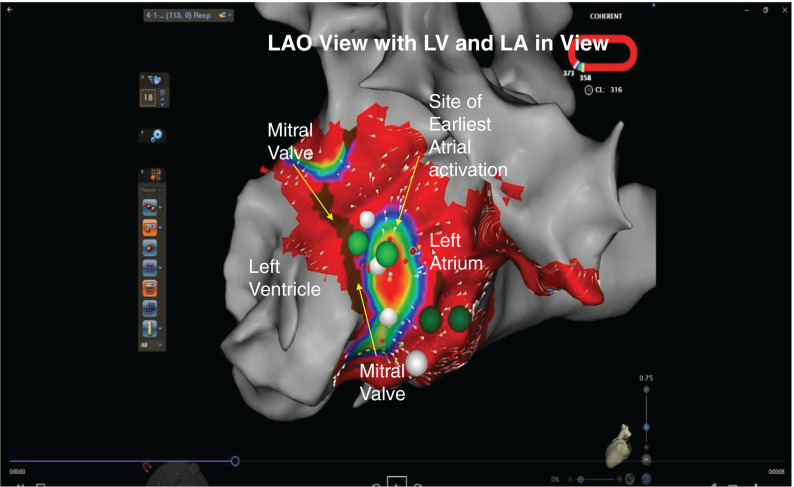
Coherent mapping demonstrating centrifugal focally appearing activation noted during mapping at the atrial exit site of an accessory pathway. LA: left atrium; LV: left ventricle; MA: mitral annulus.

**Figure 4:**
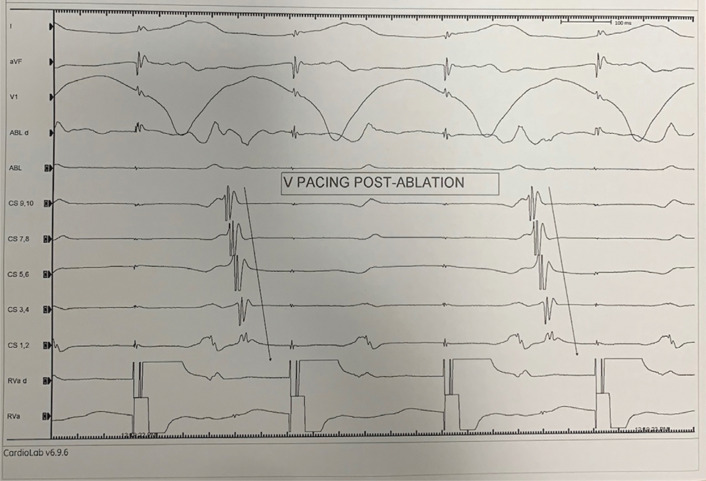
Ventricular pacing demonstrating the loss of retrograde conduction over the accessory pathway postablation.

Coherent mapping was used and the atrial exit site was mapped during ventricular pacing and during AVRT. Upon acquisition of a coherent activation map, the earliest atrial exit site showed a point source and centrifugal activation appearing as a focal tachycardia mechanism **(Figure 3 and Video 1)**. This focal appearing exit site with centrifugal spread was ablated using a 3.5-mm irrigated-tip force-sense catheter in a power-controlled mode using 35 W of power, with immediate loss of retrograde conduction through an accessory pathway **(Figure 4)**. The ablation was delivered for one minute initially, followed by 30 seconds of insurance burn. Ventricular pacing performed postablation revealed the loss of retrograde conduction. The procedure was performed without the use of any fluoroscopy.

## Discussion

Accurate identification of the atrial exit site of concealed accessory pathway conduction during AVRT can be difficult using conventional electrogram annotation. EAM using an activation map has been extensively used for both focal and reentrant arrhythmias. By estimating delays between adjacent sites relative to the fiducial point, activation map computation can be completed.

The role of activation maps using color-coding and EAM is limited during ablation of the accessory pathway. Coherent mapping is a feature of the CARTO^®^ 3 system (Biosense Webster, Diamond Bar, CA, USA) that improves the representation of electric-wave propagation over the chamber being mapped by means of coloring and direction vectors, focusing on the displayed path of cyclic arrhythmia propagation. This algorithm uses the local activation time values of all the points in a map and performs an iterative calculation to discern a global best-fit solution in these arrhythmias, analogous to calculating a regression line to show how scattered points indicate a global trend.^3^ The mechanism of the AVRT is reentry and mapping only one limb of the tachycardia circuit would yield a centrifugal pattern of activation and may help to precisely locate the target for ablation. There is also a potential for demonstrating the pathway with a slant by mapping other (ventricular) sites as well.

Although the new coherent mapping supports the mapping and ablation of complex micro- and macro-reentry circuits, we present its utility in less complex arrhythmias with normal cardiac tissue. By integrating vector and velocity information with coherent mapping, such not only provides the direction of activation but also helps to identify the critical isthmus. This may be particularly helpful in minimizing the amount of ablation that is needed to successfully and safely^4^ terminate complex atrial arrhythmias following atrial fibrillation ablation. Thus, coherence mapping has the potential to reduce procedural times and the risk of complications.

The assumption was that the atrial exit wavefront from the atrial insertion site of the accessory pathway would behave as a focal source and exhibit a centrifugal spread from a point source in the case of an orthodromic reciprocating tachycardia. Coherent mapping may therefore facilitate precise localization for ablation in a zero-fluoroscopy environment. In addition, in situations where retrograde conduction over an accessory pathway is lost because of mechanical damage, pathway bump, or incomplete ablation, the coherent map has the potential to allow for the ablation of the previously acquired and color-coded earliest activation point of the atrial exit. By precisely locating the targets, coherent mapping may hold a role in the ablation of sites close to the atrioventricular node. By accurately localizing the area of interest and providing information about the directionality of the activation, this mapping approach may help to ensure safe and effective ablation.

## Conclusion

Coherent mapping can accurately identify and localize the atrial exit of orthodromic reentrant tachycardia for precision-guided ablation in a zero-fluoroscopy environment.

## Figures and Tables

**Video 1:** Coherent mapping demonstrating a centrifugal focal appearing activation noted during mapping at atrial exit site of an accessory pathway.
